# Serum Dioxin Concentrations and Age at Menopause

**DOI:** 10.1289/ehp.7820

**Published:** 2005-03-24

**Authors:** Brenda Eskenazi, Marcella Warner, Amy R. Marks, Steven Samuels, Pier ario Gerthoux, Paolo Vercellini, David L. Olive, Larry Needham, Donald G. Patterson, Paolo Mocarelli

**Affiliations:** ^1^School of Public Health, University of California at Berkeley, Berkeley, California, USA; ^2^School of Public Health, University at Albany, Albany, New York, USA; ^3^Department of Laboratory Medicine, University of Milano-Bicocca, School of Medicine, Hospital of Desio, Desio-Milano, Italy; ^4^Department of Obstetrics and Gynecology, Mangiagalli Hospital, University of Milan, Milan, Italy; ^5^Department of Obstetrics and Gynecology, University of Wisconsin Medical School, Madison, Wisconsin, USA; ^6^Division of Laboratory Sciences, National Center for Environmental Health, Centers for Disease Control and Prevention, Atlanta, Georgia, USA

**Keywords:** 2,3,7,8-tetrachlorodibenzo-*p*-dioxin, Cox proportional hazards, dioxin, endocrine disruptors, menopause, Seveso, TCDD

## Abstract

2,3,7,8-Tetrachlorobenzo-*p*-dioxin (TCDD), a halogenated compound that binds the aryl hydrocarbon receptor, is a by-product of numerous industrial processes including waste incineration. Studies in rats and monkeys suggest that TCDD may affect ovarian function. We examined the relationship of TCDD and age at menopause in a population of women residing near Seveso, Italy, in 1976, at the time of a chemical plant explosion. We included 616 of the women who participated 20 years later in the Seveso Women’s Health Study. All women were premenopausal at the time of the explosion, had TCDD levels measured in serum collected soon after the explosion, and were ≥ 35 years of age at interview. Using proportional hazards modeling, we found a 6% nonsignificant increase in risk of early menopause with a 10-fold increase in serum TCDD. When TCDD levels were categorized, compared with women in the lowest quintile (< 20.4 ppt), women in quintile 2 (20.4–34.2 ppt) had a hazard ratio (HR) of 1.1 (*p* = 0.77), quintile 3 (34.3–54.1 ppt) had an HR of 1.4 (*p* = 0.14), quintile 4 (54.2–118 ppt) had an HR of 1.6 (*p* = 0.10), and quintile 5 (> 118 ppt) had an HR of 1.1 (*p* = 0.82) for risk of earlier menopause. The trend toward earlier menopause across the first four quintiles is statistically significant (*p* = 0.04). These results suggest a nonmonotonic dose-related association with increasing risk of earlier menopause up to about 100 ppt TCDD, but not above.

2,3,7,8-Tetrachlorobenzo-*p*-dioxin (TCDD), a halogenated compound that binds and activates the aryl hydrocarbon receptor, is a byproduct of numerous industrial processes including waste incineration (Zook and Rappe 1994). Dioxin, a known human carcinogen [International Agency for Research on Cancer (IARC) 1997], is also thought to disturb the reproductive and endocrine systems (Birnbaum and Tuomisto 2000).

Studies in rats and monkeys suggest that TCDD may affect ovarian function directly or indirectly via the pituitary (Gao et al. 2000; Li et al. 1995a; Moran et al. 2001). *In utero* and lactational TCDD exposure in rats has been associated with reduced ovarian weight and decreased number of corpus lutea and pre-antral and antral follicles (Heimler et al. 1998). Postnatal TCDD exposure in rats has been related to reduced ovarian weight gain, ovulation rate, and number of follicles as well as inhibition of follicular rupture (Gao et al. 1999; Roby 2000), morphologic changes in the ovary, and altered cyclicity with disruption of the estrous cycle (Kociba et al. 1976; Li et al. 1995a, 1995b; Roby 2000; Son et al. 1999). Although TCDD does not increase apoptosis of follicles (Heimler et al. 1998), it slows follicular maturation (Mattison 1980; Silbergeld and Mattison 1987). Postnatal TCDD exposure in monkeys has been associated with decreases in serum estradiol and progesterone, leading to anovulation in some cases (Allen et al. 1979; Barsotti et al. 1979). In a study of mature female macaques, a single TCDD dose (below the maximum in the current study) led to long-term effects on ovarian function (Moran et al. 2001).

Menopause, the cessation of menstruation, is thought to be caused by a loss of primordial ovarian follicles, resulting in the decline in estradiol production and the concomitant increase in circulating concentrations of follicle-stimulating hormone (FSH) (Lopez et al. 2000). The age of onset of menopause is believed to reflect the rate of atrophy of the ovarian follicles. Alterations in age at menopause can have important health implications because women with early menopause are at higher risk for osteoporosis, cardiovascular disease, and reproductive cancers (Bradsher and McKinlay 2000; Karagas et al. 2000).

There is limited epidemiologic evidence that endocrine-disrupting chemicals affect the natural timing of menopause. Data from a case–control study of breast cancer in North Carolina (USA) show that women with serum dichlorodiphenyldichloroethene (DDE) levels in the upper 10th percentile had an earlier onset of natural menopause than did women with levels below the median [hazard ratio (HR) = 1.4], but poly-chlorinated biphenyl (PCB) levels were not related to age at menopause (Cooper et al. 2002). Women from the Yu-cheng, China, cohort who ingested cooking oil contaminated with PCBs and polychlorinated dibenzofurans did not differ from unexposed women in their mean age at menopause or in the percentage of women who had experienced menopause (Yu et al. 2000). Among Michigan (USA) women who had been exposed to polybrominated biphenyls (PBBs) and PCBs in 1973, no association was found between serum concentrations of PBBs or PCBs and time to menopause (Blanck et al. 2004). Amenorrhea was noted in a case report of a 30-year-old Austrian woman with extremely high levels of serum TCDD (144,000 ppt) (Geusau et al. 2001). Additional evidence for the potential effects of TCDD on the menstrual cycle is our earlier report of longer menstrual cycles in exposed women who were premenarcheal at the time of exposure (Eskenazi et al. 2002).

In this study, we examine the relationship of TCDD and age of onset of natural menopause in a population of women residing near Seveso, Italy, in 1976, at the time of a chemical plant explosion. These women were exposed to the highest levels of TCDD known in residential populations (Mocarelli et al. 1988).

## Materials and Methods

### Study population.

The Seveso Women’s Health Study (SWHS) is the first comprehensive epidemiologic study of the reproductive health of a female population exposed to TCDD. Women eligible for SWHS were 1 month to 40 years of age in 1976, had resided in one of the most highly contaminated areas (zone A or B), and had adequate stored sera collected soon after the explosion (Eskenazi et al. 2000). Recruitment took place from March 1996 through July 1998. Of 1,271 eligible women, 17 could not be contacted, and 33 had died or were too ill to participate. Of the 1,221 women contacted, 981 (80%) agreed to participate. For this analysis, we included the 616 women who had not reached natural or surgical menopause before 10 July 1976, the date of the explosion, and who were at least 35 years of age at the time of interview.

### Procedure.

Details of the study are presented elsewhere (Eskenazi et al. 2000). Briefly, after informed consent was obtained, women were interviewed by a trained nurse-interviewer who was blind to TCDD level and residence of the woman. Information was collected during the interview about demographic characteristics, personal habits, and occupational, menstrual, reproductive, and medical histories. Subsequently, women were asked to undergo a gynecologic examination with a transvaginal ultrasound and to complete a menstrual cycle diary for 3 months. Medical records were abstracted for all gynecologic treatments or conditions.

### Serum TCDD laboratory analyses.

Details of serum sample selection are presented elsewhere (Eskenazi et al. 2000). The TCDD concentration in these samples was measured by high-resolution mass spectrometry methods (Patterson et al. 1987). Values are reported on a lipid-weight basis in parts per trillion by dividing TCDD on a whole-weight basis by total serum lipid content, estimated from measurements of triglycerides and cholesterol (Akins et al. 1989).

We measured TCDD in sera collected between 1976 and 1977 for 564 women, between 1978 and 1982 for 28 women, and between 1996 and 1997 for 24 women whose earlier samples had insufficient volume. For nondetectable values (*n* = 71), a serum TCDD level equal to one-half the detection limit was assigned (Hornung and Reed 1990). For women with detectable post-1977 TCDD measurements (≥10 ppt), the TCDD exposure level was back-extrapolated to 1976 using the first-order kinetic model (Pirkle et al. 1989) for women who were > 16 years of age in 1976 (*n* = 40) or the Filser model (Kreuzer et al. 1997) otherwise (*n* = 1). For the 7 women with post-1977 measures whose TCDD levels were < 10 ppt, measured values were used. The study median serum sample weight was 0.65 g, and the median limit of detection was 18.8 ppt, lipid-adjusted.

### Definitions of menopause.

Each woman was categorized by menopausal status using the following definitions: premenopause, if the woman was still menstruating or if she had amenorrhea due to pregnancy or lactation at the time of interview with evidence of subsequent menstruation from the menstrual diary or exam; natural menopause, if the woman had ≥12 months of amenorrhea not due to other obvious causes such as pregnancy, lactation, and medical conditions [World Health Organization (WHO) Scientific Group 1996); surgical menopause, if the woman had a medical-record–confirmed hysterectomy and/or a unilateral or bilateral oophorectomy; impending menopause, if the woman menstruated within 12 months, but not in the 2 months before interview or exam, whichever was most recent, and if her amenorrhea could not be explained by pregnancy, lactation, or other medical conditions; perimenopause, if the woman did not menstruate within the last 2 months before interview or exam but either gave evidence of subsequent menstruation in her menstrual diary or on ultrasound exam her endometrial lining was classified as secretory, indicating ovulation and impending menses, or if the woman reported cycles becoming less predictable (either irregular or longer) in the previous 2–5 years (a woman was not classified as perimenopausal if she reported a return to a regular cycle, if there was evidence only for a single irregular cycle, or if the irregularity was attributable to another cause); and other menopausal status, if the woman’s menopausal status could not be determined because of current oral contraceptive (OC) or other hormone use (including hormone replacement therapy) or previous chemotherapy for cancer.

### Statistical analyses.

We considered serum TCDD levels both a continuous (log_10_ TCDD) and a categorical variable based on quintiles of serum levels near the time of the explosion (1, < 20.4 ppt; 2, 20.4–34.2 ppt; 3, 34.3–54.1 ppt; 4, 54.2–118.0 ppt; 5, > 118.0 ppt). To evaluate the relationship between serum TCDD and age at natural menopause, we performed Cox proportional hazards analyses with the robust method of calculating the variance-covariance matrix (Lin and Wei 1989). The Cox model assesses effects on age-specific probabilities of reaching natural menopause by the relative hazard, or HR, the ratio of probabilities computed for each categorized level of exposure versus the reference group (< 20.4 ppt) or for the effect of a 10-fold increase in TCDD (log_10_ TCDD). Scaled Schoenfeld residuals were generated for the final multivariate model and used to test the proportional hazard assumption (i.e., that the HR is proportional over time) (Grambsch and Therneau 1994). Analyses were performed using STATA (release 8.0; Stata Corp., College Station, TX, USA). All *p*-values are two-tailed.

Covariates were considered for the multivariate Cox proportional hazards analysis if they had been reported in previous literature to be related to age at menopause. Covariates were kept in the multivariate model if they were statistically significant (*p* < 0.10) or if they changed the regression coefficient for TCDD exposure by > 10%. We considered the following as potential covariates: current body mass index (BMI), premenopausal smoking history, education, marital status, current physical activity, age at menarche, parity, OC use, and history of heart disease. We also created a variable for premenopausal history of medical conditions that could potentially be related to age at menopause, including type 1 diabetes (*n* = 1), rheumatoid arthritis (*n* = 4), radiation for cancer (*n* = 4), epilepsy (*n* = 2), hyperthyroid (*n* = 10), and untreated hypothyroid (*n* = 2) (Dorman et al. 2001; Klein et al. 2001; Steinkampf 1990). In addition to controlling for these conditions with an indicator variable, we repeated our models excluding women with these conditions (*n* = 23). The results were similar; therefore, we present only the results including the women with other medical conditions. One covariate (education) was found to violate the proportional hazards assumption (*p* = 0.06); therefore, estimates stratified by education were obtained.

For women who met the definition of natural menopause, age (in years) at last menstrual period was assigned as their age at menopause. Surgically menopausal women were censored at the age at which they had surgery. Premenopausal and perimenopausal women were censored at their age at interview. Women using OCs or other hormones and those with a history of chemotherapy were censored at the age at which they began use or treatment. Each woman was entered into the analysis at 35 years of age, before which natural menopause was unlikely to occur. Twenty-seven women (4%) were censored before 35 years of age because of surgical menopause (*n* = 7), OC use (*n* = 19), or other hormone use (*n* = 1).

We reran the final models including as menopausal the 13 women in the impending menopause group who may have been menopausal but had not yet reached the definition of natural menopause (12 months of amenorrhea). To assess the possibility that TCDD exposure is associated with conditions that would lead to surgical menopause or that it is associated with a longer menopausal transition, we also reran the final models with a redefined outcome including surgical menopause, perimenopause, impending menopause, and natural menopause.

The final models were also rerun considering alternative TCDD exposure scenarios including cumulative exposure dose (area under the curve measured in parts per trillion-years) and estimated exposure dose at time of failure or censorship (parts per trillion). These doses were estimated for each year of exposure (time dependent) following the first-order kinetic model assuming a half-life of 9 years for TCDD (Pirkle et al. 1989). Ages at risk before the explosion were assigned to the “unexposed” category. We also reran the final models excluding the 24 women for whom it was necessary to estimate TCDD exposure by back-extrapolation from TCDD levels measured in serum collected in 1996.

## Results

Characteristics of the 616 women in the analysis are presented in Table 1 for all women and by menopausal category. The mean (± SD) age at interview of the 616 women was 47.8 ± 8.1 years and ranged from 35 to 63 years. A total of 260 women (42.2%) were in pre-menopause, 169 women (27.4%) were in natural menopause, 83 women (13.5%) were in surgical menopause, 13 women (2.1%) were in impending menopause, 33 (5.4%) were in perimenopause, and 58 (9.4%) were assigned other status [current OC use (*n* = 39), other hormone use (*n* = 17), chemotherapy (*n* = 2)]. The mean (± SD) age at menopause for those in natural menopause (*n* = 169) was 49.2 ± 3.7 years (median, 49; range, 39–57), which was older than those in surgical menopause (42.7 ± 6.2 years; median, 43; range, 22–52).

All women were Caucasian, about half had less than the required amount of education, about 40% were overweight or obese (≥25 kg/m^2^), two-thirds had never smoked, about half had ever used OCs, and nearly all had been married and were parous (Table 1). Compared with premenopausal women, natural menopausal women were less educated and more likely to be overweight or obese, to be nonsmokers, to have used OCs for a shorter period of time, and to have had more pregnancies.

For each menopausal category, the median lipid-adjusted serum TCDD level and inter-quartile range (IQR) are presented in Table 2. Overall, the median lipid-adjusted serum TCDD level for the 616 women was 43.7 ppt (IQR, 24–95 ppt; range, 2.5–6,320 ppt). For premenopausal women, the median serum TCDD level was 43.6 ppt (IQR, 21–91 ppt), and for naturally menopausal women the median was 45.8 ppt (IQR, 28–100 ppt). Serum TCDD levels did not vary significantly across the menopausal categories (analysis of variance for log_10_ TCDD, *p* = 0.87).

In Cox proportional hazards modeling, the unadjusted HR associated with a 10-fold increase in TCDD (log_10_ TCDD) was 1.02 [95% confidence interval (CI), 0.8–1.3; test for trend, *p* = 0.89] (Table 3). That is, there was a 2% nonsignificant increase in risk of onset of menopause with a 10-fold increase in TCDD (e.g., from 10 to 100 ppt). After controlling for education, parity, duration of OC use, and “other medical conditions,” the association with log_10_ TCDD remained nonsignificant (HR = 1.06; 95% CI, 0.8–1.4). However, when a square term in log_10_ TCDD was added to the continuous variable model, it was statistically significant, suggesting a curvature in the dose–response curve (results not shown).

**Figure 1:**
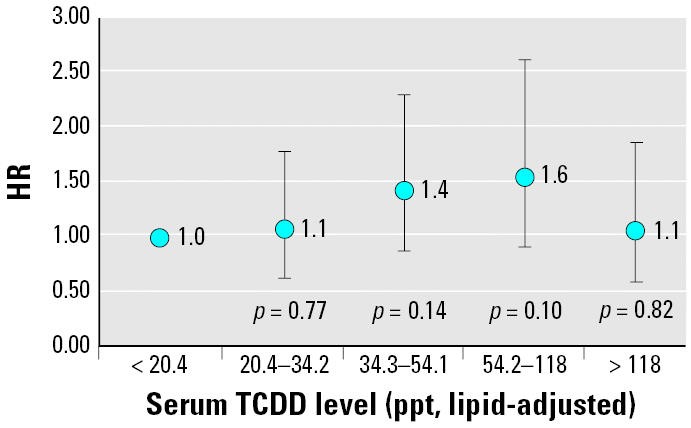
Serum TCDD quintiles and age at natural menopause: HRs and 95% CIs, SWHS, Italy, 1996–1998 (*n* = 616), adjusted for education, parity, duration of OC use, and other medical conditions. Test for trend across quintiles: quintiles 1–5: *p* = 0.44; quintiles 1–4: *p* = 0.04.

When serum TCDD levels were categorized into quintiles, risk of earlier menopause trended upward in the first four quintiles but not in the highest quintile in the unadjusted (Table 3) and adjusted models (Figure 1). After adjusting for covariates, relative to women with TCDD levels in the lowest quintile (< 20.4 ppt), women with TCDD levels in quintile 2 (20.4–34.2 ppt) had a 10% increase in hazard of natural menopause (adjusted HR = 1.1; 95% CI, 0.7–1.8; *p* = 0.77), women with TCDD levels in quintile 3 (34.3–54.1 ppt) had a 40% increase in hazard of natural menopause (adjusted HR = 1.4; 95% CI, 0.9–2.3; *p* = 0.14), and women with TCDD levels in quintile 4 (54.2–118 ppt) had a 60% increase in hazard of natural menopause (adjusted HR = 1.6; 95% CI, 0.9–2.6; *p* = 0.10). Women in the highest quintile (5, > 118 ppt), however, had only a 10% increase in hazard of earlier natural menopause (adjusted HR = 1.1; 95% CI, 0.6–1.9; *p* = 0.82). Although no increasing trend of earlier natural menopause was observed across the five quintiles (*p* = 0.44), a significant trend to earlier natural menopause across the first four quintiles was found (*p* = 0.04). Furthermore, when we excluded the 24 women who had back-extrapolated TCDD levels from 1996, the association is strengthened. Compared with women in the lowest quintile (< 20.4 ppt), women in quintile 2 (20.4–34.2 ppt) had an HR of 1.2 (*p* = 0.5); quintile 3 (34.3–54.1 ppt) had an HR of 1.6 (*p* = 0.08); quintile 4 (54.2–118 ppt) had an HR of 1.7 (*p* = 0.05); and quintile 5 (> 118 ppt) had an HR of 1.2 (*p* = 0.5) for risk of earlier menopause. The trend toward earlier menopause across the first four quintiles is statistically significant (*p* = 0.02).

The results did not change when women in the impending menopause category were classified as menopausal in the analysis (data not shown). Similar results were found when women in surgical menopause and peri-menopause were also combined with natural and impending menopause as one outcome (data not shown).

When TCDD exposure was extrapolated to the time of failure or censorship, the results were no different. Cumulative TCDD exposure (parts per trillion-years), however, was not related to age at onset of menopause (adjusted HR = 1.02; 95% CI, 0.8–1.3).

In the final models described above, nulliparity was associated with earlier natural menopause (adjusted HR = 1.9; 95% CI, 1.1–3.4), and history of OCs for at least 5 years was associated with later natural menopause (adjusted HR = 0.5; 95% CI, 0.3–1.1). We observed a nonsignificant earlier natural menopause for women who were current smokers (adjusted HR = 1.2; 95% CI, 0.8–1.7). BMI, however, was not associated with age at natural menopause.

## Discussion

The results of this study of women residing in Seveso, Italy, in 1976, at the time of a chemical plant explosion that resulted in very high levels of TCDD exposure, suggest a nonmonotonic dose-related association of TCDD levels in sera collected near the time of exposure with earlier onset of natural menopause; the trend for increasing risk is observed with TCDD levels up to about 100 ppt, but not above. Our finding is supported by the earlier mean age of menopause observed in our study (49.2 ± 3.7 years) relative to that (mean = 50.9 years) reported in an Italian clinic-based study of > 4,300 menopausal women during the same time period (1995–1997) (Meschia et al. 2000). It is also earlier than the mean age of 49.9 years reported contemporaneously for menopausal women from another unexposed province in the Lombardia region (Celentano et al. 2003).

To our knowledge, no previous epidemiologic studies have examined the relation of TCDD exposure and age at menopause. However, amenorrhea was observed in a case report of an Austrian woman with extremely high levels of serum TCDD (144,000 ppt) (Geusau et al. 2001). Our findings are also consistent with findings from a case–control study of breast cancer in women residing in North Carolina (Cooper et al. 2002). In that study, investigators did not find a relationship between age at menopause with serum levels of total PCBs (including dioxin-like and non-dioxin-like PCBs) but did find an elevated risk (HR = 1.4) for earlier menopause in women with serum levels in the top decile of DDE compared with women with levels below the median. However, the mechanism of action for TCDD is not the same as for DDE (Mizuyachi et al. 2002), and the effects of TCDD may differ depending on the estrogen-target material (Chaffin et al. 1996).

The potential impact of TCDD exposure on age at menopause is biologically plausible, as animal studies indicate (Kociba et al. 1976; Li et al. 1995a, 1995b; Roby 2000; Son et al. 1999). In a rat model, a serum estradiol concentration 8–10 times higher than normal was needed to overcome TCDD-blocked ovulation, including restoration of the luteinizing hormone and FSH surges. This suggests that the hypothalamic–pituitary axis may be less sensitive to estrogen in TCDD-treated animals (Gao et al. 2001).

If TCDD exposure induces earlier menopause, it is unlikely to occur via oocyte apoptosis. Recent data in mice suggest that TCDD does not induce *Bax* gene expression in oocytes, which is necessary for the oocyte loss related to premature ovarian failure (Matikainen et al. 2001). Although this relation remains to be examined in human cells, the findings on *Bax* activation would suggest that TCDD exposure may not cause premature ovarian failure (J. Tilly, personal communication).

We observed an inverted U-shaped relationship between TCDD serum levels and earlier menopause. An inverted U-shaped dose response has been hypothesized by Kohn and Melnick (2002) as a plausible outcome with endocrine-disrupting chemicals. Myers et al. (2003) hypothesized that at lower “physiologic” doses a chemical may mimic a hormone, but at higher doses the toxic effect of the chemical may overwhelm the stimulatory or inhibitory effects. Empirical data from animals exposed to a variety of estrogenic xenobiotics (Rubin et al. 2001; vom Saal et al. 1995) support this theory, although only one prior study of TCDD (Markowski et al. 2001) has demonstrated non-monotonic effects (i.e., of *in utero* exposure on adult weight of offspring). The present results as well as those in animals suggest a reevaluation of the presumed monotonic dose–response relationships with exposure to endocrine-disrupting chemicals that are typically tested in statistical modeling of epidemiologic data.

This study has some limitations. One limitation is the retrospective recall of age of natural menopause. However, previous studies have reported moderately high reliability and accuracy based on interview (Colditz et al. 1987). Further, in the women with surgical menopause, the reported age at menopause was similar to the age recorded in the medical record. In addition, we augmented our classification using ultrasound, menstrual diary and medical record information. We also counted women who had evidence of impending menopause as menopausal, and saw a similar pattern of results.

Although smoking has been associated with earlier menopause in a number of studies (Brambilla and McKinlay 1989; Brett and Cooper 2003; Bromberger et al. 1997; Cooper et al. 2002; Gold et al. 2001; Meschia et al. 2000; Palmer et al. 2003; Sowers and La Pietra 1995; van Noord et al. 1997; Willett et al. 1983), we did not observe a significant relationship in the present study of a TCDD-exposed population. This lack of association may be due to the paucity of heavy smokers, or possibly related to an interaction between different ligand-activated receptor pathways (Klinge et al. 2000). Another reason for the lack of association may be that we defined smoking status as that at interview. Smoking status at the time of the outcome (if it occurred before the interview) may have been different.

Another limitation of the study is that the lowest TCDD exposure group (≤20.4 ppt) experienced relatively high serum levels compared with the contemporary levels we have reported for this area (~ 2 ppt) (Warner et al. 2004). Also, although the explosion resulted in exposure specifically to TCDD, pooled serum samples collected in 1976 from females who resided in the unexposed area showed substantial background exposure to other polychlorinated dibenzo-*p*-dioxins and PCBs during this time period [90 ppt dioxin toxic equivalents (TEQ), on average, for this age group) (Eskenazi et al. 2004)]. Therefore, individuals with TCDD levels < 20 ppt might still have had substantial dioxin TEQ exposure. Because we could consider only TCDD in this study, our results may underestimate an effect due to dioxin TEQ exposure.

An advantage of this study is that we were able to measure TCDD levels in individual serum samples collected near the time of exposure, and there was a wide range of exposure. For the few women whose samples were of inadequate volume, we used serum collected between 1996 and 1997. If we exclude these women, the relation is strengthened. We have examined multiple exposure scenarios including exposure soon after the explosion as well as exposure extrapolated to each age at risk.

In summary, we observed a nonmonotonic dose–response relationship between serum TCDD levels and age of onset of natural menopause. The women in this study experienced substantial TCDD exposure during the postpubertal–adult developmental period. Animal evidence suggests that *in utero* and lactational TCDD exposure may have significant effects on ovarian follicles (Heimler et al. 1998); therefore, continued follow-up of the younger women in the SWHS cohort as well as the female offspring of the SWHS cohort is essential.

## Figures and Tables

**Table 1:** Distribution of select characteristics [*n* (%)] by menopausal status, SWHS, Italy, 1996–1998 (*n* = 616).

Characteristic	All women*^a^*	Premenopausal*^b^*	Natural menopause*^b^*	Surgical menopause*^b^*	Impending menopause*^b^*	Perimenopause*^b^*	Other*^b^*
Menopausal status	616 (100)	260 (42.2)	169 (27.4)	83 (13.5)	13 (2.1)	33 (5.4)	58 (9.4)
Age at interview [years (mean ± SD)]	47.8 ± 8.1	41.9 ± 4.7	56.6 ± 3.7	52.1 ± 6.4	51.5 ± 2.8	47.1 ± 3.2	41.8 ± 6.6
Education							
Less than required	341 (55)	85 (25)	139 (41)	59 (17)	11 (3)	26 (8)	21 (6)
Required/university	275 (45)	175 (64)	30 (11)	24 (9)	2 (1)	7 (3)	37 (13)
Current BMI (kg/m^2^)							
< 18.5	13 (2)	8 (62)	3 (23)	0 (0)	0 (0)	1 (8)	1 (8)
18.5–24.9	353 (57)	170 (48)	80 (23)	37 (10)	6 (2)	18 (5)	42 (12)
25.0–29.9	180 (29)	63 (35)	60 (33)	31 (17)	6 (3)	9 (5)	11 (6)
≥30	70 (11)	19 (27)	26 (37)	15 (21)	1 (1)	5 (7)	4 (6)
Cigarette smoking							
Never	419 (68)	155 (37)	130 (31)	66 (16)	10 (2)	25 (6)	33 (8)
Former	88 (14)	47 (53)	16 (18)	9 (10)	0 (0)	3 (3)	13 (15)
Current	109 (18)	58 (53)	23 (21)	8 (7)	3 (3)	5 (5)	12 (11)
Total OC use (years)							
0	332 (54)	111 (33)	132 (40)	53 (16)	6 (2)	18 (5)	12 (4)
< 1–5	184 (30)	106 (58)	30 (16)	21 (11)	2 (1)	11 (6)	14 (8)
≥5	100 (16)	43 (43)	7 (7)	9 (9)	5 (5)	4 (4)	32 (32)
Ever married							
No	16 (3)	11 (69)	2 (13)	1 (6)	0 (0)	1 (6)	1 (6)
Yes	600 (97)	249 (42)	167 (28)	82 (14)	13 (2)	32 (5)	57 (10)
Parous							
No	49 (8)	32 (65)	8 (16)	5 (10)	0 (0)	1 (2)	3 (6)
Yes	567 (92)	228 (40)	161 (28)	78 (14)	13 (2)	32 (6)	55 (10)

a No. (%) of column.

b No. (%) of row.

**Table 2:** Distribution of serum TCDD levels near the time of explosion by menopausal category at interview, SWHS, Italy, 1996–1998 (*n* = 616).

Category	No. (%)	Serum TCDD [median ppt (IQR)]
Premenopause	260 (42.2)	43.6 (21–91)
Natural menopause	169 (27.4)	45.8 (28–100)
Surgical menopause	83 (13.5)	43.4 (28–98)
Impending menopause	13 (2.1)	43.8 (24–105)
Perimenopause	33 (5.4)	36.5 (22–85)
Other	58 (9.4)	39.6 (17–85)
Total	616 (100.0)	43.7 (24–95)

**Table 3:** Serum TCDD levels, percentage with natural menopause, and unadjusted HRs (95% CIs) for onset of menopause, SWHS, Italy, 1996–1998 (*n* = 616).

TCDD (ppt)	*n*_mp_/*n*_tot_ (%)	HR (95% CI)
Continuous		
log_10_ TCDD	169/616 (27)	1.02 (0.8–1.3)
Quintiles		
< 20.4	24/123 (20)	1.0 (reference)
20.4–34.2	35/123 (28)	1.1 (0.7–1.8)
34.3–54.1	41/123 (33)	1.4 (0.9–2.3)
54.2–118	37/124 (30)	1.6 (1.0–2.7)
> 118	32/123 (26)	1.0 (0.6–1.8)

*n*_mp_, number of women who reached natural menopause; *n*_tot_, total number of women.
